# Editorial: Mathematical problems in physical fluid dynamics: part I

**DOI:** 10.1098/rsta.2021.0056

**Published:** 2022-06-13

**Authors:** D. Goluskin, B. Protas, J.-L. Thiffeault

**Affiliations:** ^1^ Department of Mathematics and Statistics, University of Victoria, Victoria, British Columbia, Canada; ^2^ Department of Mathematics and Statistics, McMaster University, Hamilton, Ontario, Canada; ^3^ Department of Mathematics, University of Wisconsin-Madison, Madison, WI, USA

**Keywords:** turbulence, transport, convection, mixing, vortex dynamics, *a priori* bounds

## Abstract

Fluid dynamics is a research area lying at the crossroads of physics and applied mathematics with an ever-expanding range of applications in natural sciences and engineering. However, despite decades of concerted research efforts, this area abounds with many fundamental questions that still remain unanswered. At the heart of these problems often lie mathematical models, usually in the form of partial differential equations, and many of the open questions concern the validity of these models and what can be learned from them about the physical problem. In recent years, significant progress has been made on a number of open problems in this area, often using approaches that transcend traditional discipline boundaries by combining modern methods of modelling, computation and mathematical analysis. The two-part theme issue aims to represent the breadth of these approaches, focusing on problems that are mathematical in nature but help to understand aspects of real physical importance such as fluid dynamical stability, transport, mixing, dissipation and vortex dynamics.

This article is part of the theme issue ‘Mathematical problems in physical fluid dynamics (part 1)’.

Even though the Navier–Stokes equations were introduced nearly two centuries ago as the main mathematical model on which fluid mechanics is based, many of the basic mathematical properties and physical implications of these equations are not fully understood. One category of especially difficult questions, which is by no means the only one, concerns parameter limits in which solutions to the governing equations exhibit extreme spatio-temporal complexity that is the hallmark of turbulent fluid flow. Understanding how this complexity arises from simpler flows has led to copious research in stability and transition, where the key challenge is to identify the most dangerous disturbances capable of triggering turbulence. At the same time, turbulent flows are responsible for transport and mixing of various quantities. One paradigmatic problem for turbulent transport is Rayleigh–Bénard convection, where the main outstanding question is how convective heat transport scales with the applied temperature gradient. Since providing definitive answers to such questions based on first principles has resisted all attempts, turbulence remains as one of the last major unsolved problems in classical physics.

In principle, one would hope that many of the aforementioned questions could be addressed by carefully analysing the Navier–Stokes equations. However, traditional analysis of nonlinear partial differential equations (PDEs) relies on mathematical estimates that are often too coarse to discern subtle details of the nonlinear and nonlocal dynamics that underlie incompressible fluid flows. On the other hand, in recent years, it has been possible to shed new light on some of the fundamental open problems in fluid mechanics by complementing rigorous mathematical analysis with other approaches, such as asymptotic analysis and numerical computations. For example, carefully designed computations allow us to verify the sharpness of existing analytical estimates for PDEs and to guide analysis by finding numerical evidence for or against various conjectures. A key breakthrough here was the realization that many questions concerning extreme behaviour can be studied by casting them in terms of suitably defined variational optimization problems. Additionally, considerable progress has been made recently by employing computational methods from the theory of dynamical systems which, among other developments, led to the discovery of several new invariant solutions in turbulent flows.

This theme issue offers a survey of new developments in the fundamental physics of fluid flow obtained using modelling, mathematical analysis, and computation. The included topics revolve around open problems in both mathematics and physics. Their resolution is also important to numerous applications in natural science and engineering. One example is the scaling of turbulent heat transport in the comparatively simple Rayleigh–Bénard model, whose understanding seems prerequisite to properly parametrizing turbulent transport in general circulation models for climate studies. Similarly, questions about energy dissipation in canonical turbulence models are closely related to the behaviour of drag in various engineering flows. Finally, understanding the transition to turbulence is likely to aid in numerous engineering applications, especially but not exclusively in aerospace engineering. These are just a few examples of application areas where further progress is contingent on addressing fundamental physical questions of the type discussed in the theme issue. Moreover, in highlighting some less traditional methods for certain problems, we hope to interest applied mathematicians and computational scientists developing such methods. Lastly, we hope to motivate mathematical analysts to study some of the theoretical questions raised by papers in the issue.

The guest editors dedicate this theme issue to our dear friend Prof. Charles ‘Charlie’ Doering. Charlie, who is well known and fondly remembered by the contributors here, helped to conceive this theme issue and was involved in early editing before his untimely passing away in May of 2021. His importance to fluid dynamics, as to several other fields, cannot be overestimated, and it is especially noticeable now that he has sadly left us. His enthusiasm and curiosity acted to galvanize turbulence studies, most of all for the Rayleigh–Bénard model that was the object of his professed ‘convection obsession’. Charlie’s accomplishments and knowledge, which were somehow both broad and deep, allowed him to be treated by several communities as one of their own, and in that way he formed an irreplaceable bridge between different cultures. He could explain to an experimentalist why they should care about Sobolev spaces, as well as help a mathematician adjust a calculation to better compare to existing data. The label of ‘irreplaceable’ is perhaps used too freely at times, but in this case it is no exaggeration.

This is Part I of a two-volume theme issue. One thread in Part I is the use of variational methods to study the parameter-dependence of turbulent mean quantities. A main mathematical approach to bounding such means *a priori* is the ‘background method’, which was pioneered in part by Charlie Doering. Here, Fantuzzi *et al*. [1] survey the background method in fluid dynamics and explain how its optimal version can be implemented numerically, while Chernyshenko [2] explains how this and other methods for bounding turbulent averages can be viewed as different versions of a single approach. Using the background method, Drivas *et al*. [3] bound heat transport in Rayleigh–Bénard convection with Navier-slip boundary conditions, while two other contributions explore how bounds can be improved for flows subject to physically reasonable assumptions in addition to the governing PDEs. This idea is pursued for Rayleigh–Bénard convection by Wen *et al*. [4] and for internally heated convection by Bouillaut *et al*. [5]. On a related note, Maity *et al*. [6] explore whether and how transitions between different large-scale flows in convection can be modelled as a Markov process.

Complementary to the above works deriving *a priori* bounds on extreme behaviour are several contributions that search for particular flows exhibiting such behaviour. Protas [7] reviews research that has used computational optimization methods to search systematically for extreme growth in the Navier–Stokes equations and related models, especially of quantities tied to possible singularity formation by conditional regularity results. Using similar computational optimization, Hefferman & Caulfield [8] identify optimal perturbations to extremize various measures of mixing in idealized two-dimensional flows. Tobasco [9] considers a disc subject to internal heating by nearly any distribution, and analytically constructs velocity fields that achieve the smallest possible thermal dissipation (at least within a logarithmic factor). Motoki *et al*. [10] compute steady flows that achieve maximal heat transport scaling for a modification of Rayleigh–Bénard convection with penetrable boundaries.

Transport in fluids is also a fundamental issue, in many ways related but in others distinct from the understanding of dynamical turbulence. Crippa *et al*. [11] examine the growth of Sobolev norms signalling the loss of regularity in transport by divergence-free vector fields. Klünker *et al*. [12] apply transfer operator techniques to the quantitative study of finite-time mixing and transport in time-periodic open flow systems. And finally, Vanneste & Young [13] decompose the Stokes drift due to waves into divergent and non-divergent parts, and investigate their relative magnitude.

## Data Availability

This article has no additional data.
